# Real-Time Operational Research: Case Studies from the Field of Tuberculosis and Lessons Learnt

**DOI:** 10.3390/tropicalmed6020097

**Published:** 2021-06-08

**Authors:** Anthony D. Harries, Pruthu Thekkur, Irene Mbithi, Jeremiah Muhwa Chakaya, Hannock Tweya, Kudakwashe C. Takarinda, Ajay M. V. Kumar, Srinath Satyanarayana, Selma Dar Berger, I. D. Rusen, Mohammed Khogali, Rony Zachariah

**Affiliations:** 1 International Union against Tuberculosis and Lung Disease (The Union), 75006 Paris, France; Pruthu.TK@theunion.org (P.T.); akumar@theunion.org (A.M.V.K.); SSrinath@theunion.org (S.S.); sberger@theunion.org (S.D.B.); 2 Department of Clinical Research, Faculty of Infectious and Tropical Diseases, London School of Hygiene and Tropical Medicine, London WC1E 7HT, UK; 3 The Union South-East Asia Office, C-6 Qutub Institutional Area, New Delhi 110016, India; 4 Respiratory Society of Kenya, Regent Court, Nairobi P.O. Box 27789-00506, Kenya; imbithi@resok.org (I.M.); Jeremiah.Muhwa@lstmed.uk (J.M.C.); 5 Department of Medicine, Therapeutics and Dermatology, Kenyatta University, Nairobi P.O. Box 43844-00100, Kenya; 6 Department of Clinical Sciences, Liverpool School of Tropical Medicine, Liverpool L3 5QA, UK; 7 The Lighthouse, Kamuzu Central Hospital, Lilongwe P.O. Box 106, Malawi; htweya@lighthouse.org.mw; 8 AIDS and TB Department, Ministry of Health and Child Care, Causeway, Harare P.O. Box CY 1122, Zimbabwe; kctakarinda@gmail.com; 9 Yenepoya Medical College, Yenepoya (Deemed to be University), Mangalore 575018, India; 10 Vital Strategies, New York, NY 10005, USA; irusen@vitalstrategies.org; 11 Special Programme for Research and Training in Tropical Disease (TDR), World Health Organization, 1211 Geneva 27, Switzerland; khogalim@who.int (M.K.); zachariahr@who.int (R.Z.)

**Keywords:** operational research, real-time operational research, tuberculosis, COVID-19, ethics, research capacity building, Malawi, Kenya, Zimbabwe, India

## Abstract

Real-time operational research can be defined as research on strategies or interventions to assess if they are feasible, working as planned, scalable and effective. The research involves primary data collection, periodic analysis during the conduct of the study and dissemination of the findings to policy makers for timely action. This paper aims to illustrate the use of real-time operational research and discuss how to make it happen. Four case studies are presented from the field of tuberculosis. These include (i) mis-registration of recurrent tuberculosis in Malawi; (ii) HIV testing and adjunctive cotrimoxazole to reduce mortality in TB patients in Malawi; (iii) screening TB patients for diabetes mellitus in India; and (iv) mitigating the impact of COVID-19 on TB case detection in capital cities in Kenya, Malawi and Zimbabwe. The important ingredients of real-time operational research are sound ethics; relevant research; adherence to international standards of conducting and reporting on research; consideration of comparison groups; timely data collection; dissemination to key stakeholders; capacity building; and funding. Operational research can improve the delivery of established health interventions and ensure the deployment of new interventions as they become available, irrespective of diseases. This is particularly important when public health emergencies, including pandemics, threaten health services.

## 1. Introduction

Over the years, the terms “operational research”, “operations research”, “implementation research”, “health systems research” and “health services research” have been used interchangeably to describe research conducted in health programmes using routinely collected data to try and effect change in policy and/or practice. Ask 20 operational research scientists to define what is meant by these types of research, and the likelihood is that you will get 20 different answers.

As a group working both independently and together over 30 years in health programmes in low- and middle-income countries, we have proposed a pragmatic definition of operational research: “the search for knowledge on interventions, strategies or tools that can enhance the quality, effectiveness, or coverage of programmes in which the research is being conducted” [1].

The premise that underpins this type of research is that we routinely collect mounds of data from all levels of the health system. These data can include daily out-patient attendances, daily in-patient admissions and discharges, data about patient screening, diagnostic services, enrolment to care, treatment outcomes and so on. The data are usually collected and collated as aggregate numbers in paper-based reports or electronic data sets and, more often than not, are stored away as reports in shelves or databases and rarely used after that. Operational research seeks to utilise these data, transform the data into useful information about how the health system works and use that evidence for decision making to improve public health, be it at the local, national or international level [2,3].

The operational research that we most frequently see being done uses secondary data that have been routinely collected over months or years and are used to produce information and evidence about disease control programmes, health services or health systems. However, operational research can be carried out using primary data. We would regard this as real-time operational research and define it more comprehensively as “any operational research involving primary data collection, periodic analysis and interpretation at regular intervals during the conduct of the study and dissemination of findings to policy makers for timely action.” This type of research is more labour intensive and demanding than research using previously available records. However, it is key to the effective implementation of proven interventions and to show how new products (for example, vaccines, diagnostics and treatments) can be introduced and deployed, ensuring they are available to everyone who needs them. Real-time operational research is not an academic exercise, but rather a formal evaluation of public health practice that needs to be firmly integrated and embedded within health service delivery.

This paper aims to illustrate the use and effectiveness of real-time operational research. Specific objectives are to: (i) focus on tuberculosis (TB) and show how four real-time operational research studies were conducted in Africa and Asia, with the findings leading to important changes in policy and practice; and (ii) consider and discuss how to make real-time operational research happen on the ground and be effective.

## 2. Four Case Studies of Real-Time Operational Research on TB

### 2.1. Incorrect Registration of New and Recurrent TB in Malawi

In 1999, the National TB Programme (NTP) in Malawi became concerned about the declining numbers of patients registered nationally as ‘recurrent TB’. The country was in the midst of amid a catastrophic human immunodeficiency virus (HIV) epidemic, and previous clinical studies done throughout sub-Saharan Africa had shown that HIV was strongly associated with recurrent TB [4,5,6,7]. The declining numbers of patients with recurrent TB in Malawi did not make sense, and the NTP was concerned that patients with recurrent TB were being misregistered as having new TB. A real-time operational research project was started after receiving ethics approval from the Malawi National Health Science Research Committee.

All 43 hospitals in the country that registered and treated TB patients were visited in each of the three regions over a few months during the NTP routine supervision schedules. All patients in the hospital registered with new TB were interviewed using a structured questionnaire, and they were asked whether they had ever had previous TB and treatment: if this was the case, the patient was recorded as having recurrent TB. Wherever possible, affirmative patient responses were verified using out-patient identity cards of previous TB treatment. At the end of each regional supervision, the data were analysed and discussed before moving to the next region to see if the simple protocol should be continued to be used or changed—in the event, no changes were made. The key findings at the end of the study are shown in Table 1 [8].

The study confirmed the NTP hypothesis that a substantial number of patients with recurrent TB were misregistered as having new TB. The mistake was significantly more common in patients with smear-negative pulmonary TB (PTB) and extrapulmonary TB than those with smear-positive PTB. This had important programmatic implications. First, not only were patients being misregistered in the country, but this incorrect information was being transmitted to the World Health Organization (WHO) and published in the annual WHO Global TB Reports. Second, anti-TB treatment at that time was different for new and recurrent TB patients, so some patients were incorrectly treated.

The NTP acted swiftly and decisively. Within three months, all NTP staff were briefed about the findings and were retrained, and new guidelines on how to properly register and treat TB patients were developed [9]. These guidelines were disseminated in-country, and the National TB Manual (which was the blueprint for TB control activities in the country) was updated.

The following year, the study was repeated using the same methodology to see if these interventions had worked. A considerable improvement was noted with large reductions in numbers and proportions of patients being misregistered [10]. The mis-registration of smear-negative PTB and extrapulmonary TB declined from 14.2% to 4.7% and from 8.8% to 0.9%, respectively. Over the next few years, recurrent TB as a proportion of all nationally registered TB patients rose from 3% in 1999 to 12% five or six years later, and this was probably a true reflection of the pattern of TB in the country at those times (source: Malawi NTP). The research led to a more accurate reporting of recurrent TB and patients receiving the correct treatment for that time.

### 2.2. HIV Testing and Adjunctive Cotrimoxazole to Reduce Mortality in TB Patients in Malawi

The advent of HIV in Malawi severely affected the NTP. Case numbers rose dramatically and treatment success, having been excellent at 90% or higher in the pre-HIV era, declined dramatically. A study of over 800 TB patients consecutively registered and treated in 1995 at a large district hospital found that 31% had died by the end of treatment [11]. In this study, HIV-positive patients had a 2.3 times higher hazard of death than HIV-negative patients.

During the whole of the 1990s, antiretroviral therapy (ART) was not available in sub-Saharan Africa. However, a randomised controlled trial in Cote d’Ivoire, West Africa, conducted between 1995 and 1998, showed that adjunctive cotrimoxazole administered to HIV-positive TB patients reduced their mortality by 48% [12]. Based on this evidence, the Ministry of Health in Malawi asked the NTP to assess whether a package of voluntary counselling, HIV testing and adjunctive cotrimoxazole (for those found HIV-positive) might reduce the high mortality in TB patients routinely registered for treatment. 

After receiving ethical approval from the Malawi National Health Science Research Committee, a real-time operational research project was started in Thyolo District, Southern Malawi, conducted jointly by Medecins Sans Frontieres and the NTP [13]. Between July 1999 and June 2020, all TB patients who started on anti-TB treatment were offered voluntary counselling and HIV testing. Those found to be HIV-positive were offered adjunctive cotrimoxazole provided there were no contraindications. Side effects were monitored clinically. Patients were followed up in the usual way on a regular monthly and quarterly basis with close supervision. There was periodic analysis of the data and regular meetings held with various stakeholders including the NTP director. The end-of treatment outcomes in this cohort (the intervention group) were compared with end-of-treatment outcomes in the cohort of TB patients registered the previous year between July 1998 and June 1999, in whom counselling, HIV testing and cotrimoxazole were not offered (the historical control group). Case fatality was the primary endpoint, and additional efforts were made to determine whether patients in each cohort who were lost to follow-up or transferred out of the district during treatment had died during the treatment period.

Of the 1061 TB patients in the intervention cohort, 91% were HIV tested, of whom 77% were HIV-positive, of whom 94% were given adjunctive cotrimoxazole. Of those receiving cotrimoxazole, 2% had reversible, non-serious, dermatological reactions. The numbers and proportions of patients in the intervention and control cohorts who died by the end of anti-TB treatment are shown in Table 2 [13]. There was a significant decrease in death for the whole intervention cohort compared with the control cohort, and this was particularly noted in those with smear negative PTB and in those registered with new TB. The number of TB patients needed to treat with counselling, HIV testing and adjunctive cotrimoxazole to prevent one death during anti-TB treatment was 12.5.

The study showed that it was feasible and safe for the NTP at the district level to implement the package of interventions, and it was effective at reducing mortality. Another real-time implementation research study with a slightly different methodology, conducted in the North of Malawi, produced almost identical results [14].

Once the studies had been completed, a Ministry of Health meeting was arranged with many stakeholders to discuss the results and the implications, which resulted in a policy of HIV testing and adjunctive cotrimoxazole being recommended for all TB patients in the country [15]. This policy was implemented and scaled up over several years and provided the framework for treating HIV-positive TB patients with ART once this treatment became available from 2004. The impact was huge. Death during anti-TB treatment in patients with smear-positive PTB decreased from 19% in 2002 to 7.5% in 2008, and this was associated with a striking increase in treatment success, which rose from 72% to 86% [15]. 

### 2.3. Screening TB Patients for Diabetes Mellitus in India

In 2007 and 2008, two systematic reviews showed that people with diabetes mellitus (DM) had a 2–3 times increased risk of developing TB than the general population [16,17]. Stakeholder meetings and further reviews of the literature led to the WHO and the International Union Against Tuberculosis and Lung Disease (The Union) launching a Framework for Collaborative Activities to Reduce the Dual Burden of TB and DM [18]. Integral to this framework was the recommendation to undertake bi-directional screening for the two diseases. At that time, how this was best done and monitored in routine health care settings was unknown. Several real-time operational research studies were therefore set up in China and India [19,20,21,22]. Overall ethical approval was obtained from The Union Ethics Advisory Group. Formal national ethics approval was deemed to not be necessary in the two countries, as these were judged to be programmatic feasibility studies, although in some cases, local ethics approval at health facility sites was obtained.

One of these studies focused on screening TB patients for DM in eight tertiary care hospitals and 67 peripheral health institutions in India [21]. After a two-day consultative meeting between the national programme managers, national experts, and representatives from the Union, WHO and the World Diabetes Foundation, the screening methodology, the recording and reporting registers and the necessary training of front-line staff were agreed upon. The human resources and costs needed for screening and supervision were found within the routine health service budget. The screening was as follows: all registered TB patients were asked whether they had DM or were on anti-DM medication. Those saying they had no DM were offered random blood glucose (RBG) testing. If RBG ≥ 110 mg/dl, patients were asked to return a few days later for a fasting blood glucose (FBG). If FBG ≥ 126 mg/dl, the patient was diagnosed as having presumptive DM and referred to diabetes services for a definitive diagnosis and enrolment to care. 

Implementation started in January 2012. During the conduct of the study, there was periodic analysis of the data, and a presentation of interim results was made to the India NTP director. Nine months after starting, in September 2012, the final results were collated and analysed. The key findings are shown in Table 3.

Of those registered for TB, 8% had a known diagnosis of DM. The screening procedures for those with no known diagnosis of DM worked well, with 95% or more of patients needing the RBG and FBG tests receiving them. Altogether, 13% of the cohort had presumptive DM (8% with a known diagnosis of DM and 5% with a new diagnosis of DM), and most of those were referred to and reached DM care services for further evaluation. 

Within a few weeks of the study completion, the findings were discussed at a large national meeting with all the stakeholders present. Following the meeting, there was a rapid national policy decision to screen all TB patients in India for DM routinely. This decision was made about eight months ahead of the scientific publication of the research findings [23]. The policy has since been translated into practice in various states in India. 

The same process of a national stakeholder’s meeting, followed by implementation, and a second national stakeholder’s meeting took place in China [19]. Six sites (five hospitals and one TB clinic) were selected. The study was regarded as a pilot project to assess the feasibility of the DM screening approach with a view to learning lessons for national scale-up. As such, upon completion of the project, the national authorities in China recommended further evaluations rather than a policy decision to change practice.

### 2.4. Mitigating the Impact of COVID-19 on TB Services in Three African Countries

In early January 2020, a new coronavirus, named severe acute respiratory syndrome coronavirus 2 (SARS-CoV-2), was linked to several atypical pneumonia cases in China. The disease caused by this new virus was subsequently named coronavirus disease 2019 (COVID-19). Spread occurred rapidly worldwide, and on 11 March 2020, the WHO declared COVID-19 to be a global pandemic. Most countries affected by COVID-19 authorised national lockdowns with restricted movements of the population to curb transmission of infection.

In high TB-burden countries, there was concern that the national lockdowns, combined with community fear of health facilities as places to contract COVID-19, would severely affect TB and HIV programme services. In the capital cities of three African countries (Kenya, Zimbabwe and Malawi), an operational research project was approved in April 2020 to assess whether a real-time monthly surveillance of TB and HIV activities instead of the usual quarterly surveillance might help to counteract the anticipated negative impact on TB and HIV services. It was hypothesised that if there were declines in case numbers or treatment outcomes, then the TB and HIV programmes could act more quickly on monthly information rather than waiting for quarterly information to reverse these trends. It was agreed that data would be collected and collated monthly using an EpiCollect5 application and reports sent monthly to the TB and HIV programme directors and all the other stakeholders included in the project.

The key objectives in each country were to collect, collate and report on specific TB and HIV-related data during the COVID-19 period (March 2020 to February 2021) and compare these data with those collected in the pre-COVID-19 period (March 2019 to February 2020) in which data were collected and collated retrospectively. Overall ethics approval was obtained from the Union Ethics Advisory Group, the Kenya Medical Research Institute, and Zimbabwe’s Medical Research Council. The Malawi National Health Science Research Committee waived the need for formal ethics approval on the grounds that this was programmatic work [24,25,26].

With respect to TB case detection, the key findings are shown in Table 4. In all three countries, the overall numbers of people presenting with presumptive PTB for investigation decreased, as did the numbers diagnosed with TB and registered for treatment.

After the initial lockdown period, which lasted several months in the three countries, measures to improve TB case detection were put in place. Using monthly comparative data with the pre-COVID-19 period, the differences in numbers of patients with presumptive and registered TB in the first 6-months of the COVID-19 period compared with the second 6-months of the COVID-19 period are shown in Table 5.

In Kenya, there were considerable improvements, despite industrial strike action in the health sector, which negatively affected the health services between November 2020 and January 2021. The interventions to improve TB case finding included: (i) integrated screening and fast-tracking of investigations for TB and COVID-19 in patients presenting with respiratory symptoms; (ii) active TB case finding in hot spots in the city; (iii) enhanced TB case finding that included screening of TB through mobile phones using a dedicated Unstructured Supplementary Service Data (USSD) dialling code, asking patients to dial into a toll-free TB screening call centre operated by health care workers and use of automated TB screening machines positioned at strategic spots in the community; (iv) active tracing of close contacts of index patients and (v) improved TB screening amongst people living with HIV [24].

In Malawi, there were modest improvements. The programme aimed to keep services running; healthcare workers were asked to inquire about TB symptoms in those attending out-patient departments proactively, and there was an active tracing of patients needing to be registered [25].

In Zimbabwe, there was a deterioration in TB case finding services. The interventions put in place included: (i) integrated screening and fast-tracking of investigations for TB and COVID-19 in patients presenting with respiratory symptoms; (ii) improved contact tracing in selected facilities; and (iii) the promotion of strict infection control practices at health facilities to encourage symptomatic patients to attend. Unfortunately, the country had widespread industrial strike action in the health sector between July and September 2020, and between December 2020 and January 2021, there were stock-outs of TB diagnostic reagents, which greatly reduced the ability to diagnose TB [26].

In all three countries, the data collection was led and monitored by country coordinators engaged explicitly for the study. They worked with a central monitoring and evaluation coordinator at The Union to put together the monthly reports for each country. In brief, two weeks after the end of each month, the TB and HIV data were collated, validated and presented in a monthly report as a series of figures, tables and narrative. These were sent to the TB and HIV programme directors, usually within a week of putting the data together, and shared with the study sites as well as all the other stakeholders involved in the project. The TB programme directors reviewed the monthly surveillance reports, which they received within four weeks of the end of the month and used the data for decision making. Cause and effect are difficult to disentangle from a study like this. Still, it is likely that timely access to data helped the programmes’ efforts to maintain services during this challenging time. 

The three studies have helped shed light on additional ways to improve TB case finding. Further innovative approaches that would benefit from real-time implementation research during the COVID-19 pandemic might include: (i) the strengthening of sputum specimen transportation to and from laboratories; (ii) the use of saliva as an alternative to sputum for diagnosing TB and COVID-19; (iii) the use of adequately equipped mobile vans with on-site Xpert MTB/RIF assays and ultraportable chest x-rays to provide diagnostic outreach; (iv) the application of digital platforms and connectivity solutions to maintain contact with patients during the lockdown periods and to ensure rapid delivery of test results for those being investigated; and iv) mobilization of TB survivors to facilitate contact tracing and active screening for TB in high-risk groups [27,28].

## 3. Making Real-Time Operational Research Happen and Ensuring It Is Effective

Real-time operational research, as discussed earlier, is more labour intensive than research based around the collection of secondary data. There are a number of issues that must be considered, discussed and agreed upon when planning and implementing such studies.

### 3.1. Ethics

A sound ethics framework must underpin the research studies, with an understanding that informed consent may be required from patients (this was deemed not to be needed in any of the four TB case studies) and the benefits and risks of any intervention may need careful assessment from a human rights perspective and the disease control programme itself [29]. In the four case studies, ethics approval was always sought, although in some cases, the National Scientific and Research Ethics Committee waived the need for a formal ethics review.

### 3.2. Research Relevance and Prioritization at the Country Level

The research must be relevant and a priority for the country and the disease control programme. The operational research studies in the four examples were all priorities for the national TB programmes. Programme directors were kept closely in touch with the data collection and analysis that occurred during the study and were all involved in the stakeholder meetings at the conclusion of the study. As such, the results were influential in shaping policy and practice on the ground and making a difference for the better.

Real-time operational research is beneficial in pandemic and epidemic situations where countries are desperate to maintain routine health services despite numerous challenges. The anticipated negative impact of COVID-19 on TB and HIV services in Kenya, Malawi and Zimbabwe was a case in point, where monthly instead of quarterly surveillance was welcomed and instituted by the disease control programmes to try and counteract the dramatic decreases in TB case detection and HIV testing. The data were also used to assess new strategies implemented to counteract the adverse effects of COVID-19 [24,25,26].

Another example is the Ebola Virus Disease (EVD) outbreak in Sierra Leone and Liberia in 2014. Operational research carried out after the EVD outbreak showed the adverse effects of EVD on the ability of the programmes to diagnose TB and provide HIV testing [30,31]. While this research provided useful information about what to do in the event of further EVD outbreaks, real-time operational research at the time of the 2014 EVD outbreak might have helped the programmes better weather the storm, as was shown for example with TB services in neighbouring Guinea [32].

### 3.3. Research That Adheres to International Standards of the Conduct and Reporting of Research 

The research, whether this is designed as a cross-sectional study, a cohort study or a case-control study, must be conducted and reported according to international standard guidelines [33]. The majority of the operational research studies that focus on quantitative data are observational designs and should follow the Strengthening the Reporting of Observational Studies in Epidemiology (STROBE) guidelines [34]. Qualitative research studies should follow the Standards for Reporting Qualitative Research (SRQR) [35] or the consolidated criteria for reporting qualitative research (COREQ) guidelines [36]. The four case studies on TB followed STROBE guidelines. Adherence to these quantitative and qualitative guidelines ensures that the research is conducted and reported to international standards and adds further credibility to the research study findings.

### 3.4. Comparison Groups

An important methodological consideration for real-time operational research is whether or not to have a comparison group. In the first case study on the misregistration of recurrent TB, the first published study had no comparison group. However, once guidelines had been developed and disseminated, the first study acted as a comparison for the second study to assess whether misregistration had decreased [8,10].

In the second case study, a package of counselling, HIV testing and adjunctive cotrimoxazole administered to a cohort of TB patients was compared with no package in a cohort of TB patients registered the previous year (defined as a historical control) [13]. The use of historical controls is acceptable, although it is important that the investigators clearly outline any differences in the cohort composition and/or standards of care of patients before and during the intervention that might affect the primary outcome.

Another way of using a control group is to select a concurrent comparison cohort against which to judge the intervention results. For example, a study was done in Malawi to assess TB treatment outcomes in a cohort of patients offered the package of HIV testing and adjunctive cotrimoxazole in Thyolo district in 2001 and results compared with the neighbouring Mulanje district during the same time where no such interventions were offered to registered TB patients [37]. There was a significant increase in treatment success and a significant decrease in mortality and other adverse outcomes in Thyolo than in Mulanje district, with findings comparable to those of the original study conducted in Thyolo district [13]. The use of concurrent controls is also acceptable, although care must be taken to match the situation of the intervention and comparison groups as close as possible.

Not all operational research studies need comparison groups. The third case study on screening TB patients for DM had no such group [21]. However, if the stakeholder committee had decided on two different screening methods, then a comparison study would have been needed.

### 3.5. Timely Collection and Sharing of Data

Consideration must be given to the timely collection, sharing and validation of data. The first two case studies from Malawi [8,10,13] and the third from India [21] used paper-based questionnaires and data collection forms for the primary data. All the investigators were in the country, and there was no problem with the sharing of data. The fourth case study, however, had investigators from several countries, and there was a need to regularly check and validate the data between the overall monitoring and evaluation coordinator based in India and the country coordinators based in Africa. Doing this with paper-based records was going to be difficult, if not impossible. In the Kenya, Malawi and Zimbabwe studies [24,25,26], an EpiCollect5 application (https://five.epicollect.net, accessed on 7 June 2021) was therefore used to collect the aggregate data and this was used in real-time to cross-check and validate data for the different variables and ensure that numbers added up. EpiCollect5 is a free application that can be downloaded to smartphones, and it was relatively easy to train all the data collectors in the country about how to use it.

### 3.6. Dissemination and Getting Research into Policy and Practice

In the four case studies, key implementers carried out the research, but the decision-makers in the Ministry of Health were involved right from the start in terms of conceptualization, methodology, analysis and interpretation of the data and the writing and editing of the final manuscript. Taking a research project from protocol to publication acts as a form of quality control, demonstrating to investigators and key decision and policymakers that the science is of a good standard and the findings reliable for making decisions [38]. The publication is important and, particularly if Open Access journals are used, allows for the widespread dissemination to national and international audiences.

Dissemination, for example, at a stakeholders’ meeting where decision and policymakers are present, is another essential part of sharing the findings and persuading people to adopt the recommended changes to policy and practice. In the first three case studies, interim stakeholder meetings with programme directors were held as the studies progressed, and importantly they were also held within a few weeks or months of the studies being completed leading to important national decisions being made long before the papers were published [10,15,23]. In the fourth case study, the data were put together each month as figures, tables and a narrative and sent as monthly reports to programme directors [24,25,26]. The monthly deadlines for submission were never missed, which allowed confidence to be built into the system, especially for trying out and monitoring new strategies.

### 3.7. Research Capacity Building

Skill and experience are needed to plan and conduct real-time operational research. Over the last 12 years, we have used the SORT IT (Structured Operational Research and Training Initiative) model to build capacity in programmatic or public health officers in low- and middle-income countries to undertake and publish operational research using routinely collected data [39]. This has been remarkably successful, resulting in many high-quality observational studies being undertaken and published in open access journals and adhering to STROBE guidelines [40].

SORT IT courses are structured with three one-week modules over a fixed relatively short period of 9–12 months, and as a result, most studies use routinely collected secondary data. More thought will be needed to adapt the SORT IT model to real-time operational research. This will require longer time periods between modules to ensure that (i) patient-centred ethics approvals are obtained in time, (ii) investigators are properly trained in the collection and validation of data and (iii) sample sizes are adequate to judge whether interventions are effective.

The fourth case example [24,25,26] also shows how capacity can be built on the ground in existing staff working on the front-line in health facilities. The Union central monitoring and evaluation coordinator trained all existing staff in Nairobi, Lilongwe and Harare about how to use EpiCollect5, useful knowledge not only for the study but also for future public health work. 

### 3.8. Funding

Funding is a key consideration. Operational research studies that use routinely collected secondary data are simple and relatively inexpensive to do. Operational research papers published through SORT IT courses, which included the capacity building and the publication charges, cost under 7000 euro per paper when conducted and led by the institutions without additional expertise costs [41].

Real-time operational research is likely to be more expensive. However, there are opportunities for funding. The Global Fund to fight AIDS, TB and Malaria in recent years has promoted the integration of operational research into country programmes during the funding process [42], and encourages real-time operational research for more effective uptake and use of proven interventions as well as research on the introduction and deployment of new products in the field when they become available. More efficient mechanisms, however, for accessing and deploying these funds are urgently needed.

### 3.9. Operational Research across the Spectrum of Disease

We have focused this perspective article on real-time operational research around TB. However, this type of research can be utilized for many other different diseases. A review of published papers through the SORT IT model between 2009 and 2018 showed that while 45% of operational research projects were on TB, the remainder were on other communicable diseases, non-communicable diseases, maternal and child health, trauma and health care emergencies and access to care [40]. The UNICEF/UNDP/World Bank/WHO Special Programme for Research and Training in Tropical Diseases (TDR) currently has a large grant from the UK National Institute for Health Research (NIHR) to undertake and build capacity in operational research around the growing threat of antimicrobial resistance (AMR). Two SORT IT courses on AMR from Africa and Asia have recently been completed with excellent publication outputs to date [43].

## 4. Conclusions

This perspective article defined the concept of real-time operational research. We selected four examples from the field of TB to show how this type of research can be implemented, with the findings in all four cases leading to important policy and practice changes. Important considerations about how to undertake and publish real-time operational research, build capacity and find the necessary funding are discussed. Disease control programmes, health services and health systems should include operational research as one of their core activities both to improve the delivery of established health interventions and to introduce and deploy new products and interventions as they become available. This is particularly important when health services are threatened by pandemics and epidemics.

## Figures and Tables

**Table 1:** Recurrent tuberculosis in patients registered in Malawi as having “new” tuberculosis.

Characteristics	Registered as New TB	Found to Have Had Previous (Recurrent) TB		OR (95% CI)	*p*-Value
	N	n	(%)		
All types of TB	1254	94	(7.5)		
Smear-Positive PTB	746	34	(4.6)	Ref	
Smear-Negative PTB	282	40	(14.2)	3.5 (2.1–5.7)	<0.001
Extrapulmonary TB	226	20	(8.8)	2.0 (1.1–3.7)	<0.05

TB = tuberculosis; PTB = pulmonary tuberculosis; OR = odds ratio; CI = confidence interval. Adapted from [8].

**Table 2:** Death in TB patients in the intervention (HIV testing and cotrimoxazole) and control groups, Thyolo District, Malawi.

Characteristics	Death [Total Enrolled on Treatment / Died / (%)]						*p*-Value
	HIV Testing and Cotrimoxazole Cohort			Control Cohort			
	Total N	Died N	Died (%)	Total n	Died n	Died (%)	
All TB patients	1061	299	(28)	925	333	(36)	<0.001
TB Type:							
Smear-Positive PTB	464	91	(20)	340	74	(22)	0.5
Smear-Negative PTB	282	105	(37)	288	140	(49)	<0.01
Extrapulmonary TB	315	103	(33)	297	119	(40)	0.05
TB Category:							
New	967	267	(28)	897	326	(36)	<0.001
Previously Treated	94	32	(34)	28	7	(25)	0.4

TB = tuberculosis; PTB = pulmonary tuberculosis; chi square tests used to calculate the *p* value. Adapted from [13].

**Table 3:** Screening of TB patients for Diabetes Mellitus in selected health facilities in India.

Screening Process for TB Patients	Number	(%)
Patients registered with TB	8269	
Known diagnosis of DM	682	(8)
Needing to be screened with RBG	7587	
Screened with RBG	7467	(98)
RBG≥110 mg/dl and needing to be screened with FBG	2838	
Screened with FBG	2703	(95)
FBG≥126 mg/dl and newly diagnosed with DM	402	(5)
Known and newly diagnosed with DM	1084	(13)
Known and newly diagnosed with DM and referred to DM care	1033	(95)
Known and newly diagnosed with DM and reaching DM care	1020	

TB = tuberculosis; DM = diabetes mellitus; RBG = random blood glucose; FBG = fasting blood glucose; adapted from [21].

**Table 4:** Numbers with presumptive PTB and registered TB in the pre-COVID-19 and COVID-19 periods in Kenya, Malawi and Zimbabwe.

Numbers Recorded with Presumptive PTB	Pre-COVID-19 March 2019–February 2020	COVID-19 March 2020–February 2021	Difference % between Pre- COVID-19 and COVID-19
Kenya	28,038	19,295	31.2% decrease
Malawi	11,271	6137	45.6% decrease
Zimbabwe	3270	1941	40.6% decrease
Numbers Diagnosed and Registered with TB	Pre-COVID-19 March 2019-February 2020	COVID-19 March 20-February 2021	Difference % between pre-COVID-19 and COVID-19
Kenya	3716	2676	28.0% decrease
Malawi	1822	1474	19.1% decrease
Zimbabwe	1078	715	33.7% decrease

PTB = pulmonary tuberculosis; TB = tuberculosis; adapted from [24,25,26].

**Table 5:** Numbers with presumptive PTB and registered TB between first 6-months and second 6-months of COVID-19 in Kenya, Malawi and Zimbabwe.

Numbers Recorded with Presumptive TB	Percentage Difference Compared with Equivalent Pre-COVID-19 Period	
	COVID-19: First 6-Months March 2020–August 2020	COVID-19: Second 6-Months September 2020–February 2021
Kenya	−53%	+5%
Malawi	−50%	−40%
Zimbabwe	−36%	−45%
Numbers Diagnosed and Registered with TB	COVID-19: first 6-months March 2020–August 2020	COVID-19: second 6-months September 2020–February 2021
Kenya	−35%	−20%
Malawi	−23%	−15%
Zimbabwe	−30%	−37%

PTB = pulmonary tuberculosis; TB = tuberculosis; adapted from [24,25,26].

## Data Availability

Not applicable.
